# The Role of Corporate Political Activity in Shaping Organizational Outcomes: A Systematic Literature Review

**DOI:** 10.12688/f1000research.169272.1

**Published:** 2025-10-06

**Authors:** Sumanggar Milton Pakpahan, Benny Hutahayan

**Affiliations:** 1 Universitas 17 Agustus 1945 - Jakarta, North Jakarta, Indonesia; 2 Universitas Brawijaya, Malang, Indonesia

**Keywords:** Corporate Political Activity, Non-Market Strategy, Corporate Social Responsibility, Firm Performance, Systematic Literature Review

## Abstract

**Background:**

Corporate Political Activity (CPA) has gained growing academic attention as a non-market strategy for influencing policy, reducing uncertainty, and enhancing firm-level outcomes. However, existing studies remain fragmented, often confined to single industries, regions, or political mechanisms, with limited cross-country comparisons and minimal exploration of digital political engagement. Moreover, no recent comprehensive synthesis has captured post-2020 developments across diverse institutional and industrial contexts, leaving gaps in understanding the broader patterns and boundary conditions of CPA effectiveness.

**Methods:**

This study conducts a Systematic Literature Review (SLR) of peer-reviewed empirical articles published between 2020 and 2025, guided by the PRISMA framework. Articles were systematically identified, screened, and synthesized from the Scopus database. Out of 589 initial articles, 20 met the inclusion criteria.

**Results:**

The findings show that CPA is primarily positioned as an independent variable affecting performance, innovation, and competitiveness. While CPA frequently generates positive outcomes, risks such as agency problems, reputational damage, and bribery emerge in weak governance environments. The review also reveals that integrating Corporate Social Responsibility (CSR) with CPA strengthens legitimacy and strategic impact, particularly in politically volatile contexts.

**Conclusions:**

Theoretically, this study contributes by mapping empirical trends, clarifying contextual boundary conditions, and proposing an integrated conceptual framework that bridges resource-based and institutional perspectives on CPA. Practically, it offers guidelines for aligning political engagement with ethical governance, enabling firms to leverage CPA–CSR integration to navigate complex institutional environments while maintaining legitimacy and stakeholder trust.

## 1. Introduction

In today’s volatile and complex global environment, firms increasingly recognize that competitive advantage is not solely secured through market-based strategies such as pricing, innovation, and operational efficiency. ^1^ They also engage in non-market strategies—intentional actions aimed at shaping political institutions, influencing regulation, and securing favorable policy outcomes. ^2^ Among these, Corporate Political Activity (CPA)—defined as “corporate attempts to influence government policy or gain direct benefits through lobbying, campaign contributions, advocacy, or informal political engagement” ^3^—has emerged as a critical field of scholarly inquiry. ^4^

Theoretical perspectives such as the Resource-Based View (RBV) position CPA as a strategic resource capable of delivering regulatory advantages and preferential access. ^3, 5^ Meanwhile, Institutional Theory highlights CPA’s role in ensuring legitimacy and compliance within socio-political environments. ^6, 7^ These frameworks explain why CPA is prominent in sectors with high regulatory exposure, policy sensitivity, and institutional uncertainty.

Over the past decade, empirical research on CPA has expanded across contexts and methodologies, examining mechanisms such as lobbying, political donations, board-level connections, and regulatory capture. ^8^ However, findings remain fragmented and inconsistent. While some studies show CPA enhancing firm performance by reducing uncertainty and facilitating government access, ^9^ others report unintended consequences such as agency problems, ^10^ reputational harm, or increased bribery in weak institutional contexts. ^11^ Moreover, recent research integrates CPA with Corporate Social Responsibility (CSR), suggesting a combined effect on legitimacy and stakeholder trust. ^12, 13^

Despite this growth, three key gaps remain: first, there is a lack of recent, systematic, and comprehensive synthesis capturing the post-2020 empirical landscape of CPA. Second, existing studies offer limited cross-contextual comparisons beyond U.S.-centric and sector-specific research, with much of the empirical work focusing on particular industries such as alcohol, ^14, 15^ sugar, ^16^ baby food, ^17, 18^ and ultra-processed foods. ^19, 20^ Third, there has been minimal exploration of emerging forms of CPA, including digital political engagement and transnational lobbying, which are increasingly relevant in today’s interconnected political economy. Given the dynamic nature of political environments and heightened public scrutiny of corporate influence, a Systematic Literature Review (SLR) is needed to consolidate empirical evidence, classify key variables, and identify emerging trends as well as theoretical gaps.

This study aims to address these issues by conducting a Systematic Literature Review of peer-reviewed empirical articles published between 2020 and 2025. Using the PRISMA method, ^21^ the review identifies and synthesizes studies that examine CPA as an independent variable and its relationship with firm-level outcomes. In doing so, the study contributes to the literature by providing an updated synthesis of the CPA field, highlighting the contextual and strategic conditions under which CPA generates value, and proposing a conceptual framework that integrates CPA with CSR within the broader domain of non-market strategy. By addressing these gaps, the study offers both theoretical clarity and practical guidance for scholars and practitioners in strategic management, corporate governance, and political economy, particularly for firms navigating increasingly complex institutional environments.

## 2. Literature review

### 2.1 Corporate political activity

Corporate Political Activity (CPA) refers to deliberate efforts undertaken by firms to influence government policy, regulatory decisions, and political processes in ways that serve their strategic interests. ^3^ These activities are not part of traditional market competition but fall within the scope of non-market strategy. Strategic actions that address the institutional and political environment in which firms operate. ^22^

CPA includes a wide range of practices, such as lobbying, political donations, corporate advocacy, building ties with policymakers, and participating in legislative hearings. ^8^ CPA can be classified into transactional activities, which are short-term and issue-specific, and relational activities, which are long-term and aim at establishing enduring political connections. ^23^

Empirical research shows that CPA can provide multiple organizational benefits. For instance, CPA can enhance firm performance by securing favorable regulatory environments, ^9^ strengthen competitive advantage through access to exclusive contracts or market protections, ^12^ and facilitate innovation by enabling access to government information and funding. ^24^ However, CPA is not without risks. Several studies warn of potential downsides such as reputational harm, regulatory backlash, or agency problems when CPA is conducted without transparency or with proper governance. ^11, 25^

### 2.2 Systematic literature review

A Systematic Literature Review (SLR) is a structured and transparent approach to reviewing existing research, aiming to identify, evaluate, and synthesize all relevant studies on a given topic. ^21^ In contrast to traditional narrative reviews, SLRs follow rigorous protocols, such as PRISMA (Preferred Reporting Items for Systematic Reviews and Meta-Analyses) to ensure replicability, comprehensiveness, and objectivity.

The application of SLR in business and management research has grown significantly, particularly in areas with diverse and fragmented literature, such as CPA. Given the cross-disciplinary nature of CPA which spans strategic management, political science, corporate governance, and public affairs, an SLR is well-suited to integrate findings, uncover trends, and identify theoretical and empirical gaps. ^26^

**Figure 1.**
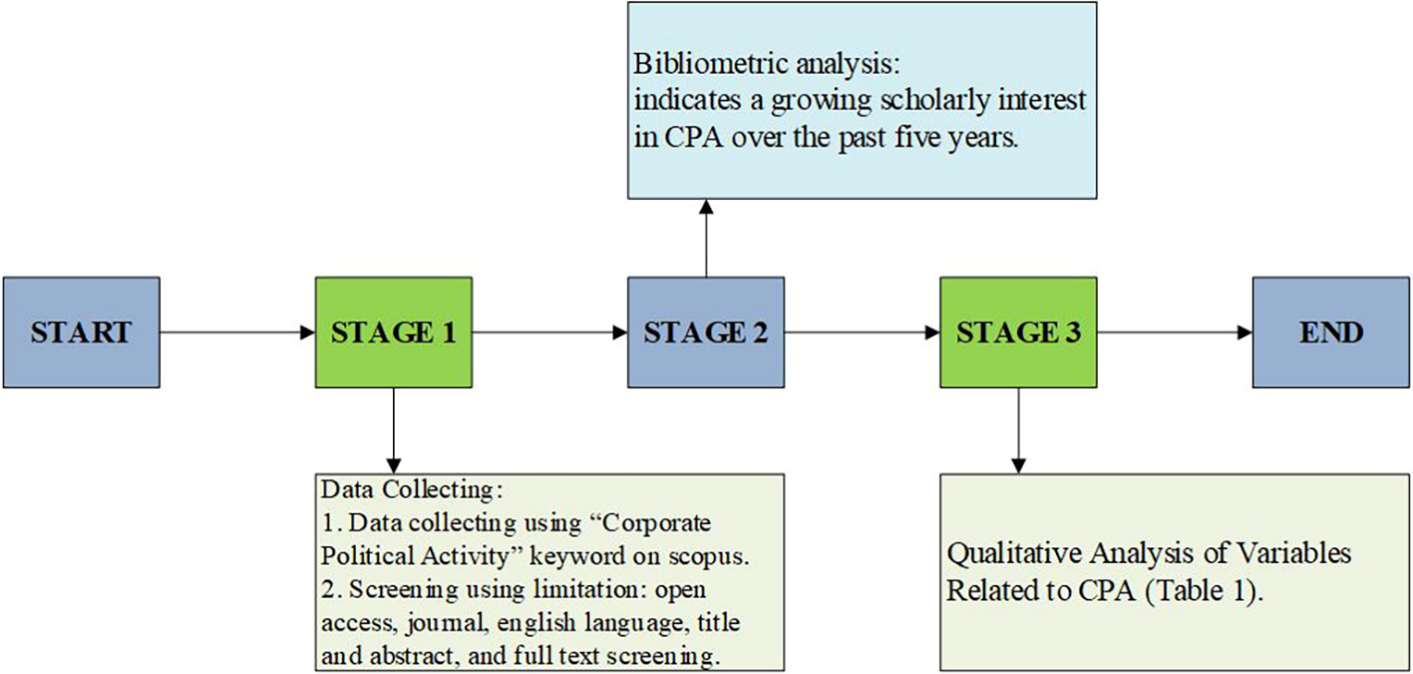
Research methodology framework.

In the context of CPA, prior reviews have either focused narrowly on specific forms (e.g., lobbying) or confined to particular regions (e.g., U.S.-based studies), limiting generalizability. Furthermore, the rapid increase in empirical studies over the past decade presents an opportunity to map how CPA has been conceptualized, measured, and linked to strategic firm outcomes such as performance and even misconduct (e.g., bribery). Figure 1 presents the stages of the systematic literature review conducted in this study.

Figure 1 illustrates the research methodology framework employed in this study, which consists of three sequential stages, beginning with data collection and concluding with the qualitative synthesis of variables related to Corporate Political Activity (CPA). The process starts with Stage 1, where relevant literature was identified using the keyword “Corporate Political Activity” through the Scopus database. During this phase, specific screening criteria were applied to refine the search results, including limiting the selection to open-access journal articles, written in English, and published between 2020 and 2025. Furthermore, the articles underwent multiple layers of screening, including title and abstract review followed by full-text assessment to ensure their empirical relevance and alignment with the research objectives.

Stage 2 involved a descriptive analysis to assess the volume and distribution of scholarly interest in CPA over the past five years. This stage served a dual purpose: first, to validate the increasing academic attention to the topic; and second, to confirm the feasibility and relevance of conducting a systematic literature review focused on CPA. The trends reinforced the rationale for further qualitative exploration by indicating a growing body of empirical research that engages with CPA from diverse methodological and theoretical standpoints.

Stage 3 consisted of a qualitative analysis of the selected articles, emphasizing the extraction and synthesis of variables related to CPA. Each article was analyzed in terms of its research method, purpose, findings, and the types of variables studied, particularly the role of CPA as an independent variable. The output of this stage is presented in Table 1 (Extended data) and forms the empirical basis for the discussion and conceptual framework proposed later in the paper. Overall, the methodological framework ensures a rigorous and structured approach in line with PRISMA standards for systematic reviews.

## 3. Methods

This study adopts a Systematic Literature Review (SLR) approach based on the PRISMA (Preferred Reporting Items for Systematic Reviews and Meta-Analyses) framework. ^21^ The PRISMA guidelines ensure a structured, transparent, and reproducible process for identifying, screening, and analyzing academic literature. The SLR process in this study involves four main steps: (1) database selection, (2) keyword search strategy, (3) inclusion criteria, and (4) study selection.

### 3.1 Data source

The Scopus database was selected as the primary source due to its comprehensive coverage of high-quality, peer-reviewed journals in the fields of business, management, and social sciences. The literature search was conducted on July 2025, to ensure the inclusion of the most recent studies.

### 3.2 Literature search strategy

This keyword was entered into the Scopus search engine without Boolean operators to capture all articles explicitly discussing CPA, regardless of the specific dependent variable or context. The search was filtered for journal articles only, excluding book chapters, conference proceedings, and editorials.

### 3.3 Inclusion criteria

To ensure consistency, relevance, and academic quality, the following inclusion criteria were applied:

IC1: Only open-access journal articles were included to ensure full accessibility for analysis.

IC2: Articles published within the last five years (2020–2025) were selected to prioritize recent research and developments.

IC3: Only articles written in English were considered to maintain linguistic consistency and facilitate comparative analysis.

IC4: Articles must contain empirical findings, relevant theoretical frameworks, and discuss CPA in relation to firm-level outcomes.

### 3.4 Study selection

The study selection process followed four main stages based on PRISMA guidelines:

1. Initial Search
  An initial search using the keyword “Corporate Political Activity” in the Scopus database yielded a total of 589 articles. No filters were applied at this stage to capture the broadest range of potentially relevant literature.
2. Screening Based on Inclusion Criteria
  The search results were screened using IC1, IC2, and IC3. After removing non-English, non-peer-reviewed, and non-open-access sources, 134 articles remained.
3. Title and Abstract Screening
  The titles and abstracts of the remaining articles were reviewed to assess their relevance to the research objectives, particularly regarding the discussion of CPA. After this screening stage, 79 articles were retained for further review.
4. Full-Text Review
  A full-text assessment was conducted on the 79 selected articles. Based on relevance to the research objectives and fulfillment of inclusion criteria, 20 articles met all inclusion criteria and were selected for final synthesis and analysis.

TITLE-ABS-KEY (“Corporate Political Activity”) AND PUBYEAR > 2019 AND PUBYEAR < 2026 AND (LIMIT-TO (OA, “all”)) AND (LIMIT-TO (DOCTYPE,“ar”)) AND (LIMIT-TO (PUBSTAGE, “final”)) AND ( LIMIT-TO (SRCTYPE, “j”)) AND (LIMIT-TO (LANGUAGE, “English”))

**Figure 2.**
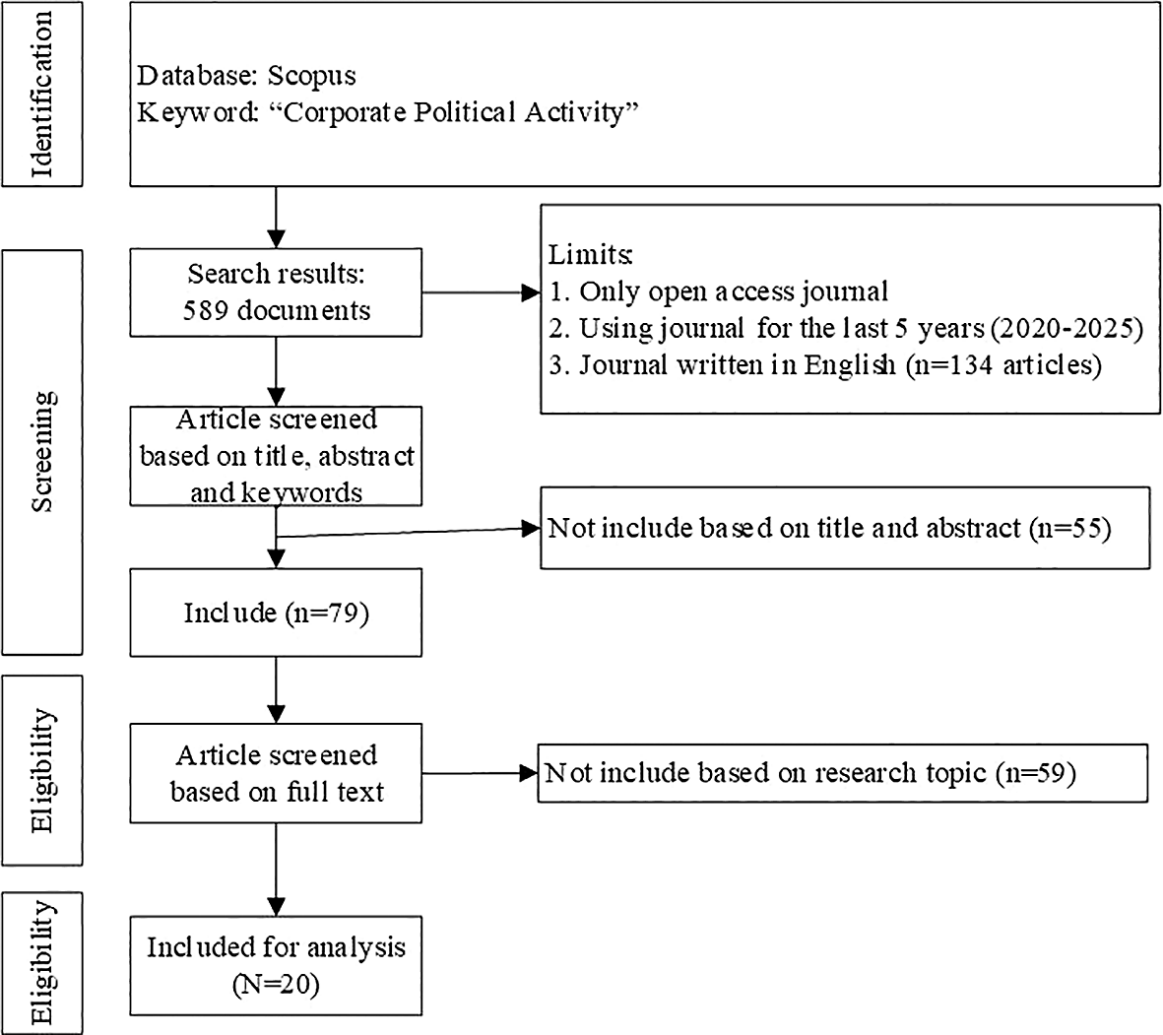
PRISMA flowchart.

PRISMA flowchart can be seen in Figure 2.

## 4. Results and discussion

**Figure 3.**
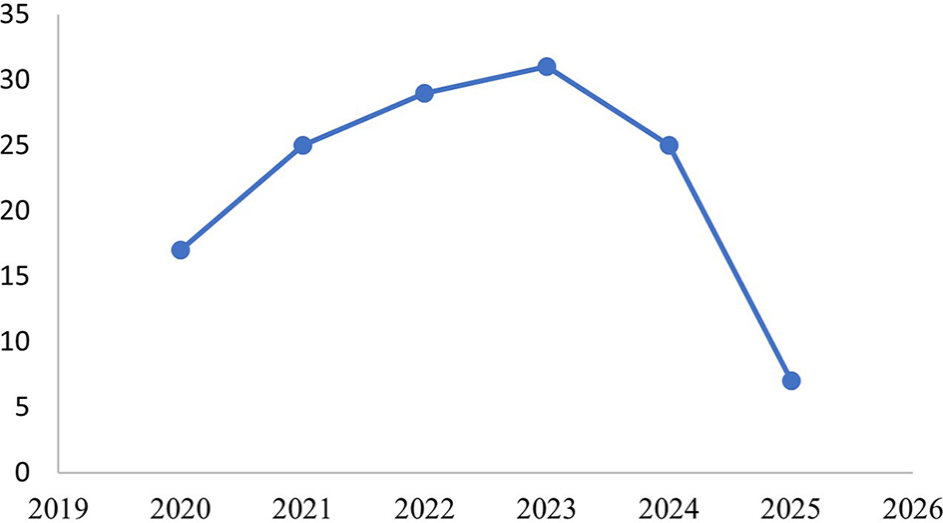
Publication trends on corporate political activity (2020–2025).

A descriptive literature overview was first conducted to understand the evolution of scholarly interest in Corporate Political Activity (CPA) over the past five years. As illustrated in Figure 3, the number of CPA-related journal articles indexed in the Scopus database increased steadily from 17 articles in 2020 to a peak of 31 articles in 2023, reflecting an upward trend in academic attention to political engagement as a strategic domain in corporate management. This growing interest may be attributed to rising global regulatory complexity, stakeholder activism, and the increasing visibility of corporate influence on public policy. While the number of publications slightly declined in 2024 (25 articles) and more sharply in 2025 (7 articles), this decline is likely a result of publication lag, as many 2025 articles were still in press or under review when the data was collected in July 2025.

This pattern confirms that CPA has evolved into a dynamic and relevant field of research, meriting a comprehensive synthesis of its empirical development. Accordingly, the following section presents the results of the systematic literature review, focusing on the characteristics of the 20 selected articles, the methods they employed, the variables investigated, and the key findings that inform the current understanding of CPA in organizational contexts.

### 4.1 Results

A total of 20 articles met the inclusion criteria and were selected for further analysis in this study. Each article underwent a systematic data extraction process to collect key information relevant to the research objectives. Using a PRISMA-aligned workflow, study-level information on research method, study purpose, main findings, and variables examined was extracted. The complete study-by-study synthesis is provided in Extended data (file name: “Table 1”) on Figshare (DOI: https://doi.org/10.6084/m9.figshare.29898380.v3). The central theme across all selected articles was Corporate Political Activity (CPA), examined either as the primary independent variable or as part of an interaction with other constructs such as firm performance, innovation, institutional development, or stakeholder perception.

This systematic literature review selected and analyzed 20 empirical studies that investigated the relationship between Corporate Political Activity (CPA) and various organizational outcomes. Table 1 (Extended data) presents a structured summary of the included articles, each selected through a rigorous screening process aligned with the PRISMA methodology. The table provides essential information on the authors, research methods, study purposes, main findings, and the variables involved. The selected studies reflect diverse methodological approaches, research contexts, and theoretical perspectives, offering a comprehensive understanding of how CPA interacts with strategic, operational, and institutional dynamics across firms and regions.

In terms of research design, all selected article adopted quantitative methods, such as regression analysis, structural equation modeling (SEM), and panel data techniques, to examine causal relationships involving CPA. For example, Li et al. ^23^ used OLS and mediation analysis to explore how organizational slack leads to CPA via entrepreneurial orientation, while Chu and Hoang ^27^ applied probit regression on a large African firm dataset to examine CPA’s dual role in facilitating and inhibiting innovation. Gounopoulos et al. ^28, 29^ employed OLS to investigate CPA’s influence on IPO transparency and SEC scrutiny.

**Figure 4.**
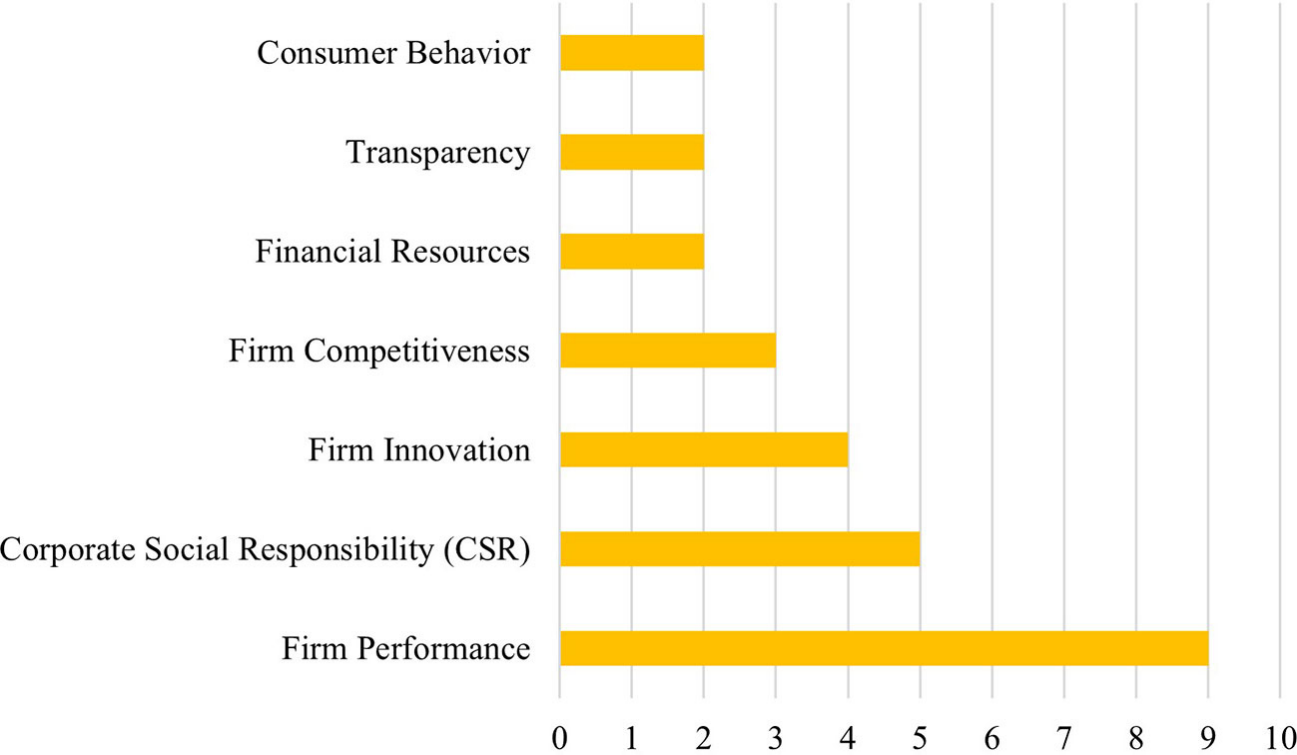
Variable frequency in CPA literature.

CPA was most frequently examined as an independent variable, often linked to outcomes such as firm performance, competitiveness, innovation, and corporate investment. Notably, firm performance appeared as the most common dependent variable ( Figure 4). Some studies, such as Hawk et al. ^30^ and Leong et al., ^31^ examined dynamic outcomes of CPA—Hawk et al. analyzed performance persistence and volatility, whereas Leong et al. assessed corporate investment and competitive advantage, finding that CPA increases investment among medium-sized and politically vulnerable firms, while large firms benefit more from R&D than from political ties. In contrast, others, like Maia et al., ^10^ explored CPA’s role in sustaining or diminishing performance persistence over time, while Liedong et al. ^11^ highlighted CPA’s darker side by documenting its positive association with firm-level bribery in weak institutional environments.

The interaction between CPA and other strategic variables, particularly CSR, emerged as a recurrent theme. Studies by Albino et al. ^13^ and Rong et al. ^32^ emphasized that CPA and CSR jointly improve performance and access to government contracts, particularly in politically volatile contexts. Furthermore, contextual variables such as institutional development, foreign ownership, political alignment, and shareholder approval were found to moderate the effects of CPA, either amplifying or constraining its strategic value.

Theoretically, most studies drew on frameworks such as the Resource-Based View (RBV), Institutional Theory, Behavioral Theory of the Firm, and Non-Market Strategy perspectives. These foundations underscore the idea that CPA functions as a resource or capability that firms leverage in pursuit of competitive advantage, risk mitigation, or regulatory leniency. In summary, the reviewed literature demonstrates that CPA is a multifaceted strategy whose impact on firm outcomes is shaped by internal capabilities, stakeholder perceptions, and external institutional conditions. While often effective in securing short-term gains or resource access, its long-term value remains contingent on alignment with broader strategic goals and ethical considerations.

### 4.2 Discussion

**4.2.1 Corporate Political Activity**

Corporate Political Activity (CPA) is consistently positioned as a strategic tool employed by firms to influence regulatory environments and secure competitive advantages. Across the 20 selected articles, CPA appears predominantly as an independent variable that affects various organizational outcomes, often interacting with contextual moderators such as political alignment, shareholder approval, and institutional quality. ^9, 29^ The studies indicate that while CPA can enhance firm legitimacy and performance, its effectiveness is contingent on how it is implemented and the institutional environment in which the firm operates. ^12, 13^ Moreover, CPA often acts as a channel to access non-market resources, but its misuse, particularly in weak institutional settings, may result in negative externalities such as increased bribery. ^11^

In internationalization contexts, lobbying—as a CPA activity—tends to promote international expansion whereas political connections can impede it, with effects conditioned by firms’ experience and product diversification. ^33^ At the performance level, CPA in isolation may depress outcomes, but greater international and product diversification enables firms to leverage CPA more effectively to improve performance. ^34, 35^ At the societal level, CPA can also erode citizens’ trust in companies and reduce perceived governance legitimacy through trust-based mechanisms. ^36^

**4.2.2 Corporate Social Responsibility**

Corporate Social Responsibility (CSR) consistently appears in the reviewed literature as an important independent variable, often examined alongside Corporate Political Activity (CPA) as part of an integrated non-market strategy. In several studies, CSR and CPA are jointly positioned as independent constructs, each contributing distinct but complementary influences on firm-level outcomes such as competitiveness, performance, and innovation.

For example, Adomako et al. ^12^ analyze the effects of CPA and CSR on firm competitiveness and find that both variables have positive and significant effects individually, while their interaction further strengthens competitiveness, especially in conditions of economic adversity. Similarly, Albino et al. ^13^ demonstrate that neither CPA nor CSR alone is sufficient to enhance organizational performance in politically unstable environments. However, when combined, the two strategies generate superior outcomes, particularly in contexts characterized by institutional weakness or regulatory uncertainty.

Rong et al. ^32^ further support this finding by showing that the CPA–CSR interaction increases a firm’s likelihood of securing government contracts. In their study, CSR acts as a signaling mechanism of legitimacy and social responsibility, while CPA functions as a tool for institutional access and influence. When used together, they produce a stronger strategic position, especially in emerging or politically fragmented markets.

In addition to their interaction, CSR is also shown to have independent effects on innovation and stakeholder perceptions. In the study by Vasquez, ^37^ CSR was compared with CPA and Corporate Social Advocacy (CSA) in shaping consumer perceptions. The findings suggest that CSR enhances credibility and positive word-of-mouth, although CPA when accompanied by motive transparency can outperform CSR in terms of reputational outcomes.

These results underscore that CSR is not merely a reputational tool, but a strategic lever that complements CPA in delivering both market and non-market outcomes. When aligned, the two variables strengthen firm competitiveness and legitimacy; when misaligned or perceived as insincere, they can undermine stakeholder trust, as highlighted by Tortosa-Edo and Lopez-Navarro. ^36^ Therefore, the results indicate that CSR, alongside CPA, functions as a core component of non-market strategy, and its effectiveness is amplified when deployed in conjunction with political engagement. This dual approach appears especially relevant in unstable institutional contexts, where firms must simultaneously navigate political influence and maintain public legitimacy.

**4.2.3 Firm performance**

Firm performance emerges as the most frequently studied dependent variable in the CPA literature, appearing in at least nine of the selected studies. However, the empirical findings demonstrate mixed and context-dependent effects, highlighting the complex nature of the relationship between CPA and firm performance.

Several studies report a positive association between CPA and firm performance under specific institutional or organizational conditions. For instance, Hoepner and Lin ^9^ find that CPA significantly improves long-term firm value, particularly when political contributions are explicitly approved by shareholders and aligned with dominant political ideologies (e.g., the Democratic Party in the U.S.). Similarly, Borges and Ramalho ^8^ show that firms with stronger political embeddedness through CPA-CSR integration tend to achieve higher financial performance within the European Union. In Leong et al., ^31^ CPA enhances corporate investment and long-term competitiveness, especially for mid-tier firms and politically vulnerable organizations. The study notes that while larger firms may have better internal capabilities, politically active mid-sized firms leverage CPA to gain access to strategic resources and reduce institutional uncertainty, leading to improved financial outcomes.

However, not all studies report favorable outcomes. Some highlight the diminishing or even negative effects of CPA on firm performance. For example, Maia et al. ^10^ reveal that while corporate lobbying is positively associated with CEO remuneration, it is negatively related to overall corporate performance. This suggests potential agency problems, where political activities benefit top executives rather than shareholders, reflecting a misalignment between CPA and firm value creation. Additionally, Shirodkar et al. ^33, 34^ demonstrate that CPA alone can have negative implications for firm performance. However, this adverse effect may be mitigated when firms possess strong international or product diversification strategies. This finding emphasizes that CPA, when not integrated into a broader strategic framework, may fail to deliver sustainable performance benefits.

A particularly critical insight comes from Liedong et al., ^11^ who examine the role of CPA in weak institutional environments. Their study of over 25,000 firms across 41 African countries finds that CPA is positively associated with the likelihood of engaging in bribery. This suggests that in the absence of strong governance mechanisms, firms may use CPA not only to influence policy but also to secure preferential treatment through informal or illicit channels, ultimately distorting market competition and undermining institutional legitimacy.

Finally, Hawk et al. ^30^ offer a longitudinal perspective by analyzing CPA’s effects across 6,000 firms in 14 democratic countries. Their findings indicate that while CPA can modestly stabilize performance over time by reducing volatility, the advantage quickly erodes due to electoral cycles and political turnover. This implies that the sustainability of CPA-driven performance gains is limited unless supported by complementary internal capabilities such as R&D and talent development.

In sum, the evidence suggests that CPA’s impact on firm performance is not universally positive. Its effectiveness depends on shareholder alignment, governance structures, firm characteristics, and institutional context. Furthermore, excessive or unethical political engagement, particularly in corruption-prone environments can backfire, harming both performance and reputation.

**4.2.4 Firm competitiveness**

Firm competitiveness is frequently identified in the reviewed studies as a strategic outcome influenced by CPA, particularly in emerging markets and politically volatile environments. CPA enhances competitiveness by enabling firms to access privileged information, shape industry-specific regulations, and secure favorable treatment from government bodies. For example, Adomako et al. ^12^ show that CPA improves the competitive positioning of firms in the Global South by mitigating external pressures such as economic adversity and leveraging internal capabilities like marketing strength. The interaction between CPA and CSR in this context is found to further amplify competitiveness, especially when firms operate under limited institutional support.

Leong et al. ^31^ add further nuance by showing that CPA disproportionately benefits mid-sized and politically vulnerable firms, which often lack internal R&D capabilities or global brand strength. For these firms, political engagement becomes a substitute for resource gaps, allowing them to gain access to investment opportunities and policy incentives that would otherwise be difficult to obtain. However, the study also notes that larger firms tend to derive less marginal value from CPA, as their competitiveness is more strongly driven by market capabilities like technological innovation or scale efficiency.

Moreover, the effectiveness of CPA in driving competitiveness appears to be contingent on strategic alignment between political tactics and firm resources. Firms that engage in CPA without clear internal integration or without legitimacy-building activities such as CSR may experience short-term gains at the expense of long-term sustainability. This suggests that CPA should be embedded within a broader non-market strategy, coordinated with ethical signaling and stakeholder engagement to sustain its competitive benefits.

**4.2.5 Firm innovation**

Firm innovation emerges as a critical yet complex outcome in the literature on CPA. Several studies recognize CPA as a mechanism through which firms can gain access to innovation-enabling resources, including government subsidies, research grants, or policy protections. For instance, Kim and Roh ^24^ find that CPA positively moderates the relationship between internal resources (such as human capital investment, international experience, and political connections) and innovation performance in Vietnamese SMEs. CPA in this context acts as a non-market facilitator that strengthens the translation of internal capabilities into innovative outcomes.

On the other hand, Chu and Hoang ^27^ highlight a more paradoxical dynamic. Their study, using data from over 7,000 firms across Sub-Saharan Africa, shows that while CPA may improve access to innovation inputs, it may simultaneously dampen intrinsic innovation motivation. Specifically, firms that heavily rely on political ties may become less incentivized to invest in organic R&D or technological development, leading to a crowding-out effect. This finding reflects the potential substitution effect between non-market and market-based strategies, particularly in resource-constrained or rent-seeking environments.

The reviewed studies collectively suggest that CPA’s impact on innovation is not inherently positive or negative, but rather depends on how it is balanced with market-based innovation drivers. When CPA is used as a supplement to internal capability-building, it can accelerate innovation outcomes by reducing regulatory barriers and improving policy access. However, when used as a replacement for innovation investment, it risks entrenching political dependency and eroding long-term competitiveness. Therefore, firms should approach CPA as a complementary strategy that supports the development of market-driven innovation.

**Figure 5.**
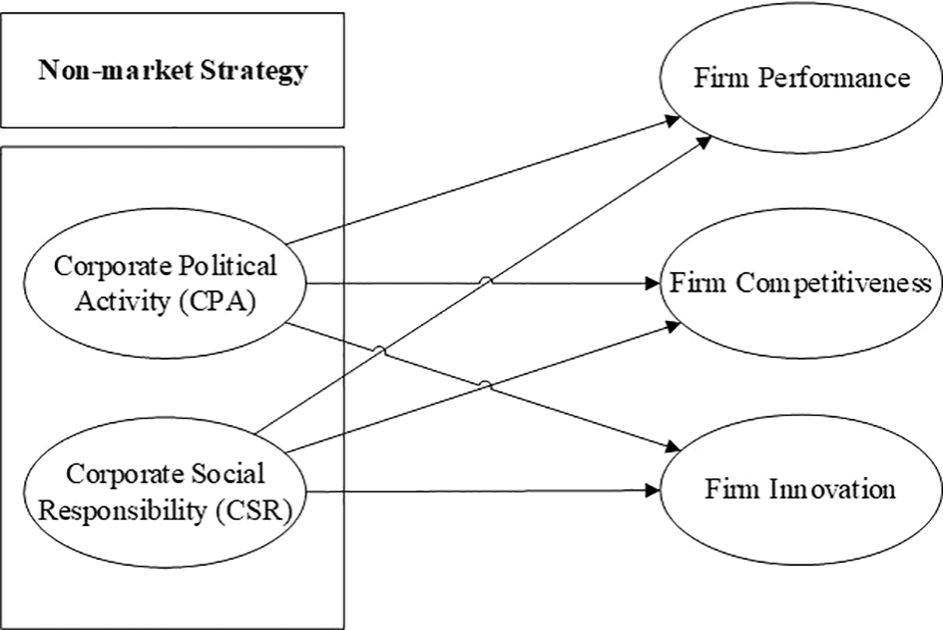
Conceptual framework.

Figure 5 shows the conceptual framework synthesized from the selected empirical studies illustrates the pivotal role of non-market strategies, particularly Corporate Political Activity (CPA) and Corporate Social Responsibility (CSR), in influencing firm-level outcomes. CPA and CSR jointly represent firm engagement beyond market-based transactions, targeting institutional, regulatory, and societal dimensions to enhance firm performance.

CPA is shown to directly affect firm performance, competitiveness, and innovation by shaping favorable policy environments, reducing regulatory uncertainty, and securing access to strategic resources. ^9, 12^ This influence is particularly salient in unstable or institutionally weak environments where formal market mechanisms are insufficient to sustain competitive advantage. ^11, 13^ The reviewed literature also suggests that CPA’s impact is contingent on internal organizational characteristics (e.g., political alignment, marketing capability) and external legitimacy factors.

Simultaneously, CSR plays a dual role as a reputational shield for politically active firms and as an independent driver of innovation and stakeholder trust. CSR enhances firm innovation by promoting long-term investment in social value and sustainability, especially when aligned with stakeholder expectations. ^24, 32^ Furthermore, the interaction between CPA and CSR often results in a compound effect, whereby CPA facilitates access while CSR justifies legitimacy, jointly improving firm competitiveness and performance. ^8, 35^

By integrating CPA and CSR into a unified non-market strategy, this framework captures a multidimensional view of firm strategic behavior. It suggests that the most successful firms are those that simultaneously engage political institutions and societal stakeholders, using CPA and CSR not as substitutes, but as complementary tools. The three firm outcomes such as: performance, competitiveness, and innovation, thus positioned as critical measures of strategic effectiveness in navigating complex business environments.

**4.2.6 Theoretical contributions**

This review makes several important theoretical contributions to the literature on Corporate Political Activity (CPA), framed through the lenses of the Resource-Based View (RBV), Institutional Theory, and Non-Market Strategy perspectives. From an RBV standpoint, the findings position CPA as a context-dependent dynamic capability that can generate competitive advantage when aligned with complementary internal resources such as marketing capability, R&D investment, and human capital. ^8, 9, 31^ Several studies demonstrate that CPA can enhance firm performance, investment, and competitiveness when these internal capabilities are present, particularly under conditions of political alignment and shareholder approval. ^9, 31^ For example, Hoepner and Lin, ^9^ Leong et al., ^31^ and Borges and Ramalho ^8^ position CPA as a valuable yet context-specific resource that yields strategic benefits when deployed in alignment with firm-specific strengths.

From the perspective of Institutional Theory, the review underscores that the effectiveness and legitimacy of CPA are contingent upon governance quality, political ideology alignment, and stakeholder norms. ^11, 36^ In strong governance environments, CPA can enhance legitimacy and policy access; in weak governance contexts, however, CPA can lead to reputational harm, bribery, and diminished stakeholder trust, as shown in Liedong et al. ^11^ and Tortosa-Edo and López-Navarro. ^36^ These findings highlight the importance of institutional alignment, ethical safeguards, and compliance systems when engaging in political activities.

In relation to Non-Market Strategy scholarship, this study advances understanding by demonstrating the synergistic role of CPA and Corporate Social Responsibility (CSR) as complementary non-market tools. ^12, 13, 32^ CPA functions as a mechanism for policy access and institutional influence, while CSR provides normative legitimacy and sustains the firm’s social license to operate. Studies such as Adomako et al., ^12^ Albino et al., ^13^ and Rong et al. ^32^ show that when deployed together, CPA and CSR can amplify strategic impact and stakeholder acceptance.

A synthesized framework of key findings, theoretical contributions, and managerial implications is provided in Extended data: Table 2 (file name: “Table 2”), on Figshare (DOI: https://doi.org/10.6084/m9.figshare.29898380.v3). These contributions enrich theoretical understanding of CPA by clarifying boundary conditions, identifying interaction effects with CSR, and highlighting risks associated with institutional weaknesses and agency problems. They also provide a conceptual foundation for future research to examine emerging forms of CPA, such as digital lobbying and transnational political engagement, and to explore their integration within broader non-market strategies. The following Conclusion section summarizes the study’s overall contributions, practical implications, and avenues for future research.

## 5. Conclusion and recommendation

This review systematically analyzed 20 empirical studies published between 2020 and 2025, mapping how Corporate Political Activity (CPA) influences firm performance, competitiveness, and innovation. The evidence confirms that CPA operates predominantly as an independent strategic lever, yet its effectiveness is highly contingent on institutional quality, political alignment, and internal governance structures.

From a theoretical perspective, the review advances the Resource-Based View by framing CPA as a context-dependent dynamic capability, the Institutional Theory by identifying governance quality and legitimacy as key boundary conditions, and Non-Market Strategy scholarship by demonstrating the synergistic role of CPA–CSR integration in enhancing both policy access and societal legitimacy.

Practically, Firms should avoid overreliance on CPA in weak institutional environments, where political engagement risks fostering bribery or undermining legitimacy. Combining CPA with CSR can strengthen stakeholder trust and enhance access to policy benefits, particularly in volatile contexts. Managers should align CPA initiatives with long-term corporate objectives, supported by transparent governance and ethical safeguards.

The review is limited to open-access, English-language, Scopus-indexed articles, potentially omitting relevant non-English or proprietary studies. Future research should examine digital lobbying, cross-border CPA strategies, and post-crisis political engagement. Longitudinal, multi-country datasets are needed to assess the sustainability of CPA-driven advantages over political cycles. In sum, CPA is a powerful yet double-edged tool: when strategically aligned and ethically executed, it can secure enduring advantage; when mismanaged, it risks eroding both firm performance and societal trust.

## Ethics and consent

Ethical approval and consent were not required.

## Data Availability

No data associated with this article. Repository name: Data Systematic Literature Review Corporate Political Activity. https://doi.org/10.6084/m9.figshare.29898380.v3 ^38^ This project contains the following extended data:
• [Data] (Collection of article data used for literature review) • [Table 1] (Data Synthesis from the 20 Selected Articles) • [Table 2] (Theoritical Contributions) Figshare: PRISMA checklist for “The Role of Corporate Political Activity in Shaping Organizational Outcomes: A Systematic Literature Review”. https://doi.org/10.6084/m9.figshare.29898380.v3 ^38^ Data are available under the terms of the Creative Commons Attribution 4.0 International license (CC-BY 4.0).
