# Supplementary material for: The Role of Corporate Political Activity in Shaping Organizational Outcomes: A Systematic Literature Review
Source: F1000Res. 2026 Apr 17;14:1046. Originally published 2025 Oct 6. [Version 2] doi: 10.12688/f1000research.169272.2 (PMC13109708; doi:10.12688/f1000research.169272.2)
Supplement: Peer review history

# Reviewer report: Reviewer response for version 2

**DOI:** 10.5256/f1000research.198371.r475973

**Published:** 2026-04-24

**Authors:** Heap Yih (John) Chong

**Affiliations:** 1 Nanjing Audit University, Nanjing, China

I have no further comments.

Are the rationale for, and objectives of, the Systematic Review clearly stated?

Yes

Is the statistical analysis and its interpretation appropriate?

Yes

If this is a Living Systematic Review, is the ‘living’ method appropriate and is the search schedule clearly defined and justified? (‘Living Systematic Review’ or a variation of this term should be included in the title.)

Not applicable

Are sufficient details of the methods and analysis provided to allow replication by others?

Yes

Are the conclusions drawn adequately supported by the results presented in the review?

Yes

Reviewer Expertise:

Social science

I confirm that I have read this submission and believe that I have an appropriate level of expertise to confirm that it is of an acceptable scientific standard.

# Reviewer report: Reviewer response for version 1

**DOI:** 10.5256/f1000research.186570.r429867

**Published:** 2025-12-11

**Authors:** Daniel J Fiorino

**Affiliations:** 1 American University, Washington, District of Columbia, USA

This is a valuable and professional article and should be indexed. The presentation was clear and capable. The article makes good use of the literature and presents the context well. The discussion of the methods is clear and accessible. The conclusions are explained clearly, and the reservations are noted. Corporate political activity is more effective if it is grounded in a strategy based on Corporate Social Responsibility. The only thoughts I would add--these are not a requirement for indexing but suggestions if the authors do any revisions--are that some attention be given to the role of third-party auditing in establishing the credibility of CSR strategies and reports and that there be slightly more discussion of areas for future research. But this is a capable review with a thorough and nuanced discussion of findings and implications. It may be indexed in its current form.

Are the rationale for, and objectives of, the Systematic Review clearly stated?

Yes

Is the statistical analysis and its interpretation appropriate?

Yes

If this is a Living Systematic Review, is the ‘living’ method appropriate and is the search schedule clearly defined and justified? (‘Living Systematic Review’ or a variation of this term should be included in the title.)

Yes

Are sufficient details of the methods and analysis provided to allow replication by others?

Yes

Are the conclusions drawn adequately supported by the results presented in the review?

Yes

Reviewer Expertise:

Environmental Policy, the effects of different policy tools on outcomes, the interaction between the public and private sectors in achieving environmental results.

I confirm that I have read this submission and believe that I have an appropriate level of expertise to confirm that it is of an acceptable scientific standard.

# Response

**Published:** 2026-04-02

**Authors:** Sumanggar Milton Pakpahan

**Affiliations:** Government Science, Universitas 17 Agustus 1945 - Jakarta, North Jakarta, Special Capital Region of Jakarta, Indonesia

This is a valuable and professional article and should be indexed. The presentation was clear and capable. The article makes good use of the literature and presents the context well. The discussion of the methods is clear and accessible. The conclusions are explained clearly, and the reservations are noted. Corporate political activity is more effective if it is grounded in a strategy based on Corporate Social Responsibility.

**Author response: **Thank you for your positive and constructive assessment. We appreciate your recognition of the article’s clarity, methodological presentation, engagement with the literature, and overall contribution. We also value your observation that corporate political activity is more effective when grounded in a Corporate Social Responsibility-based strategy, which is consistent with the central synthesis and conclusions of this review.

 The only thoughts I would add--these are not a requirement for indexing but suggestions if the authors do any revisions-are that some attention be given to the role of third-party auditing in establishing the credibility of CSR strategies and reports and that there be slightly more discussion of areas for future research. But this is a capable review with a thorough and nuanced discussion of findings and implications. It may be indexed in its current form.

**Author response:** Thank you for this constructive and encouraging comment. We appreciate your suggestion regarding the role of third-party auditing in strengthening the credibility of CSR strategies and reports, as well as your recommendation to expand the discussion of future research. In response, we have added a clarifying discussion in the CSR section to emphasize the importance of credible external verification for reinforcing legitimacy. We have also expanded the future research discussion in the conclusion section, particularly by outlining directions related to digital political engagement, cross-border CPA strategies, and quantitative hypothesis-driven research based on the synthesized conceptual framework.

# Reviewer report: Reviewer response for version 1

**DOI:** 10.5256/f1000research.186570.r421664

**Published:** 2025-10-11

**Authors:** Heap Yih (John) Chong

**Affiliations:** 1 Nanjing Audit University, Nanjing, China

This is a well-structured and timely systematic review. The methodology is clear, and the focus on post-2020 literature fills a genuine gap. Below are my comments for the paper:

 - The review relies exclusively on the Scopus database and uses a single, specific keyword: "Corporate Political Activity." Some explanations should be added in the conclusion section. 

 - While a conceptual framework is mentioned (Figure 5), it is not well articulated. 

 - The conclusion briefly mentions future research areas like "digital lobbying" and "cross-border CPA strategies." Given that this was identified as a gap in the introduction, these avenues deserve more elaborate and specific guidance.

Are the rationale for, and objectives of, the Systematic Review clearly stated?

Yes

Is the statistical analysis and its interpretation appropriate?

Yes

If this is a Living Systematic Review, is the ‘living’ method appropriate and is the search schedule clearly defined and justified? (‘Living Systematic Review’ or a variation of this term should be included in the title.)

Not applicable

Are sufficient details of the methods and analysis provided to allow replication by others?

Yes

Are the conclusions drawn adequately supported by the results presented in the review?

Yes

Reviewer Expertise:

Social science

I confirm that I have read this submission and believe that I have an appropriate level of expertise to confirm that it is of an acceptable scientific standard, however I have significant reservations, as outlined above.

# Response

**Published:** 2026-04-02

**Authors:** Sumanggar Milton Pakpahan

**Affiliations:** Government Science, Universitas 17 Agustus 1945 - Jakarta, North Jakarta, Special Capital Region of Jakarta, Indonesia

The review relies exclusively on the Scopus database and uses a single, specific keyword: "Corporate Political Activity." Some explanations should be added in the conclusion section. 

**Author response: **Thank you for this valuable comment. We have added a clarification in the conclusion section to acknowledge that the review relies on the Scopus database and the specific keyword “Corporate Political Activity” as methodological limitations. We have also included a recommendation for future research to expand database coverage, broaden keyword combinations, and test the identified relationships through quantitative hypothesis-driven studies.

 While a conceptual framework is mentioned (Figure 5), it is not well articulated. 

**Author response:** Thank you for this helpful comment. We have addressed this point by creating a separate subsection (4.2.6) for Figure 5 and expanding its explanation. The revised text now clarifies the analytical purpose of the framework, the conceptual roles of CPA and CSR, and the rationale for highlighting firm performance, competitiveness, and innovation as the main synthesized outcomes.

 The conclusion briefly mentions future research areas like "digital lobbying" and "cross-border CPA strategies." Given that this was identified as a gap in the introduction, these avenues deserve more elaborate and specific guidance.

**Author response: **Thank you for this valuable comment. We have expanded the conclusion section to provide more specific future research guidance. In particular, we now elaborate on research directions related to digital political engagement and cross-border CPA strategies, and we also link these directions to the conceptual relationships synthesized in Figure 5 to highlight opportunities for future quantitative hypothesis-driven research.

 Sincere appreciation is extended for the thoughtful and constructive comments, which have been highly valuable in strengthening the manuscript.
